# Reported experience of patients with single or multiple chronic diseases: empirical evidence from Italy

**DOI:** 10.1186/s12913-018-3431-0

**Published:** 2018-08-23

**Authors:** Milena Vainieri, Cecilia Quercioli, Mauro Maccari, Sara Barsanti, Anna Maria Murante

**Affiliations:** 1 Laboratorio Management e Sanità, Institute of Management, Scuola Superiore Sant’Anna, Piazza Martiri della Libertà, 33, 56127 Pisa, Italy; 2 Azienda USL Toscana Sudest, Siena, Italy; 3 Azienda USL Toscana Nordovest, Pisa, Italy

**Keywords:** Chronic care model, Patient satisfaction, Multi-morbidity, Monitoring system, Self-management

## Abstract

**Background:**

More and more countries have been implementing chronic care programs, such as the Chronic Care Model (CCM) to manage non-acute conditions of diseases in a more effective and less expensive way. Often, these programs aim to provide care for single conditions instead of the sum of diseases. This paper analyzes the satisfaction and better management of single and multiple chronic patients with the core elements of chronic care programs in Siena, Italy. In addition, the paper also considers whether the CCM introduced in Siena has any influence on satisfaction and better self-management.

**Methods:**

Survey data from patients with single chronic (*N* = 500) and multiple chronic diseases (*N* = 454), assisted by the Local Health Authority in Siena (Tuscany, Italy), were considered for the analysis. Variables on education, monitoring system, proactivity, relational continuity, model of care (CCM versus no CCM) and patient demographics were used to detect which strategies are associated with a higher patient-reported ability to better self-manage the disease and overall patient satisfaction. Logistic and ordinary logistic models were executed on data related to patients with both single and multiple chronic diseases.

**Results:**

The results showed that monitoring was the sole strategy associated with overall satisfaction and better self-management for both single and multiple chronic patients. Relational continuity also showed a significant positive association with better self-management perception for both patient groups, but had a positive association with patient satisfaction only for single chronic patients. Enrolment in the CCM was not associated with both overall satisfaction and better management for the two patient groups.

**Conclusions:**

Strategies that are significantly associated with satisfaction and perception of better disease self-management were the same for both single and multiple chronic patients. The delivery of care based on the Siena CCM does not seem to make a difference in the perception of better self-management and overall satisfaction for all the patients. Other concurrent strategies implemented by the regional government in Tuscany on primary care monitoring and health promotion could partially explain why CCM does not have a significant influence.

## Background

The increasing ageing population and the high impact on healthcare expenditure of chronic patient care are important issues for national and local governments. Some countries have been implementing disease management programs (DMPs) in order to effectively and efficiently take care of chronic patients. In general, DMPs are based on specific components, such as the integration of community resources, self-management support, delivery system redesign, decision support systems, clinical information systems, and organizational support. Among these components, self-management is considered an essential factor of chronic care treatment [1] because it significantly impacts on the quality of patients’ daily lives, on their physical and mental well-being [2], their active participation in self-monitoring and/or decision making processes [3], by improving patients’ knowledge and skills [4, 5]. DMPs have been found to have positive results in terms of patient health status and resources [6]. The Chronic Care Model (CCM) and its adaptations are examples of proactive disease management approaches that aim to manage the non-acute/chronic conditions in a more effective and less expensive way [7, 8].

However, DMPs, including the CCM, are still far from the person-oriented care [6, 9–12] and do not meet all the multifaceted requirements of comorbidity at the level of the individual [13]. This may be a critical issue if we consider that the trend of comorbidity in the ageing population is increasing [14–16] and a positive association has been found between comorbidity, health care use and costs across healthcare systems [17]. Providing care to patients with multiple chronic conditions requires a comprehensive and focused-person approach, which means considering episodes of care as part of the life-course instead of taking care of single diseases [11, 12].

Although some authors found that that patients with single chronic condition were, on average, less satisfied with their care than patients with two or more chronic illnesses [18, 19], evidence on patient satisfaction with care and their perception on self-management is still limited and controversial [6, 20].

The paper investigates how CCM strategies (i.e. education, delivery system and data monitoring system) affect satisfaction and self-management as perceived by patients with single or multiple conditions, both those who are part of a CCM and those who are not.

### Framework of analysis

**Fig. 1:**
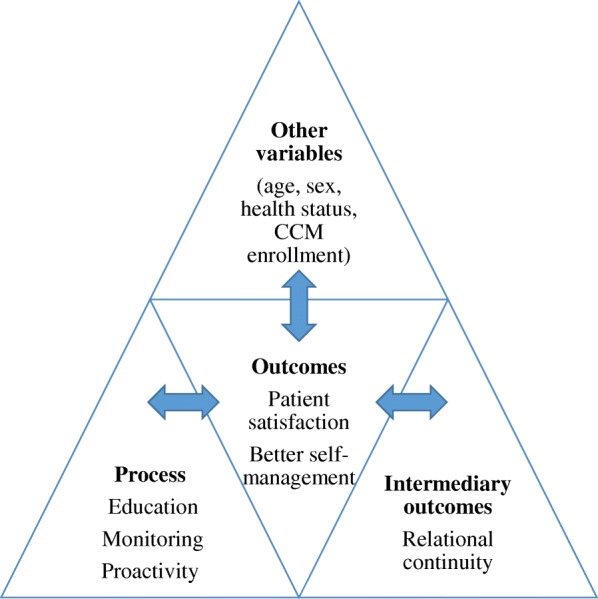
Framework of analysis

To analyze the differences in satisfaction and better self-management of patients with single or multiple chronic diseases, we considered the most recurrent elements characterizing the CCM, which was the inspiration behind the model set up in Siena [11, 21]. We grouped CCM domains into three levels of care [22]: i) process; ii) intermediate outcomes; and iii) outcomes (see Fig. 1).

#### Process

The process refers to the core activities and tools implemented to support physicians and patients in better managing the disease. These include education, proactivity strategies, and monitoring system.

##### Self-management support (*Education*)

Educational programs aimed at developing self-management skills and abilities appear to be the most common drivers of positive outcomes in chronic diseases management. These include initiatives to promote healthy lifestyles, how to deal with pain, drugs, stress etc. Such initiatives may consist in a single meeting to educate patients by general practitioners (GPs), nurses or other figures, or in small group workshops using structured formats [23, 24]. Noël et al. [25] observed that patients with multiple chronic conditions are willing to receive more information in comparison with single disease patients, hence their education can require a multi-perspective and multi-specialist approach [25].

##### Delivery system design *(Proactivity)*

This domain focuses on teamwork, planned visits and continuous follow-ups. Generally, the type and number of follow up visits are defined by the disease guidelines, and personalized according to the patient’s profile. Several studies provide substantial evidence that particular delivery actions improve care, such as expanding the care team to include office nurses and tracking core components of care with telephone follow-ups [22].

##### Decision support system (*Monitoring)*

The integration of evidence-based guidelines within daily clinical practice should enable physicians to monitor the clinical parameters that are required within the clinical guidelines. In general, reviews demonstrate that decision support systems are often successful, although the magnitude of effect may be modest and may also improve provider performance more than patient health measures [26].

#### Intermediary outcome

The health outcomes of chronic disease management are mostly expected after a medium-long period. Intermediate outcomes are related to how the physicians have been building patient loyalty, and in turn the continuity in care [27]. In this study we focused on continuity of care.

##### Relational continuity

Relational continuity is part of the larger concept of continuity of care and consists of “the longitudinal relationship between primary care providers and patients, in terms of accommodation of patient’s needs and preferences, such as communication and respect for patients” [28]. Some authors [29] consider relational continuity as a core value of primary health care. There are several aspects and definitions of relational continuity [29], and in this study we refer to the concept of accumulated knowledge that is included in the Components of Primary Care Index developed by Flocke [30]. When follow-up information is noted down during visits, patients perceive that physicians are aware of their medical history over time.

#### Outcomes

The outcomes of an effective chronic disease program can include the patient’s health status, the patient’s ability to self-manage the burden of disease in their daily life, and the overall patient satisfaction.

##### Overall satisfaction

In literature, patient satisfaction is defined as an outcome of health care provision [31–33]. It is expected that all the above actions should lead to a higher overall satisfaction with the physician, although findings are inconclusive [6].

##### Better Self-management

Improving self-management can lead to positive changes in the outcome of disease management (health and satisfaction) and generally in the patients’ quality of life. Patients that report higher levels of self-efficacy in managing the burden of disease within their daily lives also have better health outcomes [24, 34].

In this work, we compared patients with single chronic conditions to patients with multiple chronic conditions, both enrolled and not enrolled in the CCM. Our aim was to find out whether and how patient satisfaction and improvements in self-management (outcomes) are associated with a) education, proactivity monitoring system (process), and b) relational continuity (intermediate outcome). We expected that education would influence the perception of better self-management, due to the existing evidence on the effectiveness of self-management education programs [8].

## Methods

### Study setting

Chronic patients involved in this study were on the GPs’ list from the LHA in Siena, Tuscany (Italy). GPs are the first contact for the most common health problems and act as gatekeepers for drug prescriptions and access to secondary and hospital care. GPs are involved in delivering various primary care services such as health promotion and preventive care, diagnoses, treatment, and the follow up of non-complex, acute, and chronic conditions. They also have a key role in coordinating services for patients with chronic diseases. GPs in Italy are not directly employed by the national health care service, but work as independent contractors.

The chronic disease program in Tuscany Region started in June 2010 and was based on CCM model principles in accordance with the LHAs [35]. Multidisciplinary teams composed of GPs and nurses, physiotherapists, dieticians, and medical specialists were created, each with specific tasks to support chronic patients [35]. Nurses were responsible for contacting patients for routine services, scheduling specialist visits, managing individual or group patient counselling, providing self-management support, and recording patient basic data (such as weight, waist circumference, blood pressure, blood glucose). The CCM program in Tuscany was set up for patients with diabetes, heart failure, hypertension, stroke and COPD.

The adoption of the CCM strategies has never been mandatory for GPs, who have voluntary joined the new model of care. GPs receive an incentive if they enrol patients in the CCM and meet related targets [36, 37].

It is also worth mentioning that some process indicators related to chronic care management (e.g. glycated haemoglobin measurement, etc.) were included within the performance evaluation system (PES) adopted by the regional government in Tuscany to manage the performance of both local health authorities and primary care providers [38]. These indicators refer to all LHAs and districts independently of the extent to which CCM is implemented. The indicators are also part of the incentive reward scheme of the general managers of the LHAs [39]. Hence, in Tuscany all care providers (LHAs, districts and GPs) were monitored and evaluated in relation to a number chronic care management indicators.

In the Siena LHA, the program started in May 2010 with diabetes and heart failure with a limited number of GPs joining the model. In October 2010 the program also included COPD and stroke. In 2014, around 40% of the population in Siena were enrolled in the CCM [40] (source http://performance.sssup.it/anteprima/admin/pages/tuscany.php?kpi=B26.1&anno=2015).

Our study considers chronic patients assisted by GPs in Siena who were and were not part of the CCM program.

### Survey

Between 2014 and 2015, a survey was administered to a sample of chronic patients assisted by 82 GPs from the Siena LHA who were responsible for a total of 95,474 patients. Patients were interviewed by trained personnel who contacted them by phone. The Siena LHA enrolled personnel from throughout the national civil service. These personnel received training to collect data from patients using both quantitative (like our questionnaire) and qualitative (other Siena LHA projects) methods. They were supervised by one of the authors of this paper (CQ).

Consensus to participate in this survey was collected during the patients’ contacts with the LHA and no ethical consensus was required in accordance with regional law on personal data treatment.

Patients answered a questionnaire of 22 questions divided into four sections: 1. Patients’ path and experience; 2. Counselling and decision support; 3. Self management Support; 4. Self reported health status.

Sections were adapted from existing questionnaires on patient experiences with primary care services [41, 42], especially those focusing on patients with chronic and multiple chronic illnesses [18, 25, 43].

### Eligibility and sample

Patients involved in the study met the following criteria: they were resident in the province of Siena; they were over 18 years old, and had either chronic heart failure or diabetes. The total number of eligible patients was 15,317. Eligible patients were classified in patients with single and multiple chronic illnesses, using information from the Tuscan Regional Health Agency, whose criteria are reported in Table 1, and from Barnett et al.’s definition of multi-morbidity [14]. In addition, these two groups were stratified into those enrolled and not enrolled in the CCM program. Hence, the respondents were randomly selected from the list of patients stratified in four groups: i) single chronic disease patients enrolled in the CCM; ii) single chronic disease patients not enrolled in the CCM; iii) patients with multiple chronic diseases enrolled in the CCM; iv) patients with multiple chronic diseases not enrolled in the CCM.

**Table 1:** Criteria applied to identify chronic patients through administrative datasets

Conditions	Hospital data (ICD9cm)	Drug data (ATC)	Exemption
Diabetes	250*	A10*	250
Heart failure	428, 3981, 40,201, 40,211, 40,291, 40,401, 40,403, 40,411, 40,413, 40,491, 40,493		428
Hypertension			000, 401, 402, 403, 404, 405
ischemic heart disease			414

*includes all subcodes associated to the main classification

The extracted randomized sample consisted of 1300 patients.

### Statistical analyses

After descriptive statistics, we performed the related tests to compare the findings of the two single and multiple chronic patient groups. We performed 4 models to analyze the two outcomes for the two groups of chronic patients: model 1 and 2 analyzed better self-management for single and multiple chronic patients respectively; model 3 and 4 analyzed the overall satisfaction for single and multiple chronic patients. The variables used into the four models are reported in Table 2.

**Table 2:** Variables used in the models

Domains of the framework	Variables	Operationalization of the variables	Variables in the statistical model
Outcomes of care	Better self - management	‘After meeting your GP, do you feel to be able to better self manage your own situation’ - (1 = yes; 0 = no)	Dependent variable model 1 and 2
	Overall satisfaction	Ordinal scale: 1 worse, 2 medium, 3 good, 4 very good.	Dependent variable model 3 and 4
Process of care	Self-management support (Education)	Sum of 6 dummy variables:1 ‘Explain how to monitor major symptoms’; 2 ‘Explain how to carry out medication’; 3 ‘Explain what to do in urgent case’; 4 ‘Explain how to control the pain’; 5 ‘Explain how to control the stress’; 6 ‘Suggest following a healthy diet’.	Independent variable model 1–4
	Delivery system design (Proactivity)	Average of two dummy variable, considering if the GP or the nurse have planned the visit or the patient has to do by his/her own	Independent variable model 1–4
	Decision support system (Monitoring)	Sum of 5 dummy variables:1. ‘during your visit GP controlled pressure’; 2. ‘during your visit GP controlled glycemic measures’; 3. ‘during your visit GP controlled weight’; 4. ‘during your visit GP controlled waist circumference’ and 5. ‘during your visit GP controlled your life style’	Independent variable model 1–4
Intermediate outcomes of care	Relational continuity of care	Sum of 3 dummy variables: “He/she knows important information about my medical background”;“He/she knows about my living situation”; “This doctor doesn’t just deal with medical problems but can also help with personal problems and worries”	Independent variable model 1–4
Other variables	CCM	1 = enrolled in CCM, 0 = not enrolled in CCM	Independent variable model 1–4
	Age	Continuous variable	Independent variable model 1–4
	Health status	Self assessment of health status from 0 to 10	Independent variable model 1–4
	Gender	1 = female, 0 = male	Independent variable model 1–4

Better self-management was a dummy variable (yes/no) while the overall satisfaction with the care received was an ordinal scale (1 = worse, 2 = medium, 3 = good, 4 = very good). Consequently, we executed logit regressions for the better management outcome and ordinary logistic regressions for the overall satisfaction.

The independent variables included in the models were: monitoring, education, proactivity, patient’s perception of their medical history being known by the medical GP (as a proxy of interpersonal continuity), and enrolment in the Siena CCM.

Monitoring refers to the sum of five dummy issues: 1. ‘during your visit the GP checked your blood pressure’; 2. ‘during your visit, the GP checked your glycemic measures’; 3. ‘during your visit, the GP checked your weight’; 4. ‘during your visit, the GP checked your waist circumference’ and 5. ‘during your visit, the GP asked questions about your life style’. Cronbach’s alpha for the five variables is 0.87.

Education is a composite score computed as the sum of six dummy issues: 1 ‘Explain how to monitor major symptoms’; 2 ‘Explain how to carry out medication’; 3 ‘Explain what to do in urgent case’; 4 ‘Explain how to control the pain’; 5 ‘Explain how to control the stress’; 6 ‘Suggest following a healthy diet’. Cronbach’s alpha for the six variables is 0.83.

Proactivity is the average of two dummy variables, considering whether the GP or the nurse planned the visit or the patient had to do it himself/herself.

Relational continuity is a composite score computed as the sum of three dummy questions “He/she knows the important information about my medical background”; “He/she knows about my living situation”; “This doctor doesn’t just deal with medical problems but can also help with personal problems and worries”. The Cronbach’s alpha for the three variables is 0.82.

The variable that measures the enrolment into the CCM is a dummy variable where 1 is the inclusion of the patient in the CCM, and 0 otherwise.

As other control variables we considered: health status (a continuous variable from 0 to 10), age (a continuous variable) and gender.

All analyses were performed using STATA 15.

## Results

The response rate was 76%. We excluded 64 observations because in these cases the member of the medical staff involved in patient care was not the GP. Hence, the final sample was made up of 500 single chronic patients and 454 multiple chronic patients.

Table 3 reports the main descriptive statistics for CCM – no CCM within single and multiple chronic patients groups for the variables considered. The chi square test for better management, gender and revealed that differences between the answers of patients enrolled in CCM and those who are not are not statistically significant (*p* > 0,05) for both the two groups of single and multiple chronic patients.

**Table 3:** Descriptives of patients with single and multiple diseases

Variables	CCM	No CCM	CCM	No CCM
Patient characteristics				
Age	74.37	72.72	74.76	73.09
Female (%)	52.19	52.52	53.28	57.3
Health Status	6.55	6.66	6.23	6.55
Process of care				
Monitoring	2.27	2.07	2.19	2.06
Proactivity	0.38	0.39	0.39	0.38
Education	3.58	3.53	3.71	3.4
Intermediary outcome				
Relational continuity	2.89	2.83	2.82	2.84
Outcomes				
Overall satisfaction (mean)	3.44	3.43	3.42	3.37
Better self-management (%)	92.47	87.77	84.96	89.76

The Mann-Whitney test reported that statistically differences between patients enrolled in CCM versus those who are not were significant only for the monitoring strategy in the group of single patients and for health status and education in the group of multiple chronic patients.

Table 4 reports the results on the two groups’ perception of better self-management. The factors significantly affecting both groups were the relational continuity and monitoring strategy. For both groups, relational continuity had a very strong effect on better management. Interestingly, participation in the CCM program did not have any statistically significant influence on better management. Neither proactivity nor education seemed to lead to the perception of better management.

**Table 4:** Results of better self management models

	Model 1 Single chronic patients		Model 2 Multiple chronic patients	
	Odds Ratio	*P*	Odds Ratio	*p*
Better Self-Management				
CCM	1.88	*0.23*	0.53	*0.12*
Monitoring	1.61	*0.04*	1.63	*0.01*
Proactivity	1.61	*0.41*	0.76	*0.53*
Education	1.2	*0.21*	0.95	*0.62*
Relational Continuity	86.67	*0*	56.36	*0*
Health Status	1.42	*0.01*	0.98	*0.9*
Age	0.99	*0.74*	0.97	*0.25*
Sex	2.3	*0.13*	0.81	*0.61*
Constant	0	*0*	0	*0.01*
N. observation	454		497	
LR chi2 (7)	162		173	
Prob > chi2	0		0	
Log likelihood	−63.46		− 100.46	
Pseudo R2	0.56		0.46	

Table 5 shows the results related to the overall satisfaction of the two patient groups. These models show that relational continuity was statistically significant only in the case of single morbidity patients with a coefficient of 2.38. The monitoring factor was statistically significant for both groups with an odds ratio of around 1.44. CCM, education and proactivity were not statistically significant in any group.

**Table 5:** Overall satisfaction models

	Model 3 Single chronic patients		Model 4 Multiple chronic patients	
	Odds Ratio	*P > |z|*	Odds Ratio	*P > |z|*
Overall satisfaction				
CCM	0.91	*0.63*	1.09	*0.61*
Monitoring	1.44	*0*	1.43	*0*
Proactivity	1.09	*0.65*	1.27	*0.22*
Education	1.01	*0.8*	1.07	*0.14*
Relational continuity	2.38	*0*	1.25	*0.19*
Health Status	1.07	*0.24*	1.04	*0.41*
Age	1.01	*0.39*	0.99	*0.98*
Sex	1.01	*0.98*	0.82	*0.29*
cut1	−4.62		−3.7	
cut2	−3.06		−1.61	
cut3	−0.97		0.26	
N. observation	443		494	
LR chi2 (7)	33.94		32.05	
Prob > chi2	0		0	
Log likelihood	− 416.26		− 482.87	
Pseudo R2	0.04		0.03	

## Discussion

The three strategies put in place by chronic care programs and the intermediary outcome represented by the relational continuity of care did not always affect the two observed outcomes (overall satisfaction and better self-management). Relational continuity for both groups, had a highly positive effect on better self-management, but a moderate association with overall satisfaction only for single chronic patients. This confirms that the accumulated knowledge of GPs in primary care plays a pivotal role in terms of patients’ perception [29]. The higher the relational continuity, the higher the probability of better self-management, as perceived by patients and also the higher overall satisfaction for single chronic patients. For both single and multiple chronic patients, education was shown not to influence better self-management and the overall satisfaction.

For multiple chronic patients, this finding is not surprising. The literature provides with different results on the topic, for instance education does not affect overall satisfaction of multiple chronic patients in Carlin et al. [19] while it does in Fan et al. [44]. This inconclusive evidence may also be explained by the complex relationship and needs of multiple chronic patients [25]. Instead, the fact that education was not associated with overall satisfaction and better self-management for single chronic patients is quite surprising. This could be explained by both the educational model chosen in Siena and how the GPs applied it. In fact, although self-management programs follow precise rules and principles [34], how these programs are applied together with other external factors, such as the GP’s role, may affect the success of these initiatives [21, 45].

The perception of better management and overall satisfaction, for both groups, was affected only by the monitoring strategy, in a positive way: the higher the perception of being monitored, the higher the probability that patients felt that they were able to manage their own situation. While other authors found that older and female chronic patients registered a higher level of satisfaction [18] in our models neither gender nor age influenced the outcome.

The study has some limitations regarding the analyses. Firstly, the missing data related to the monitoring strategy. We imputed the missing values using the mean score for single and multiple chronic patients. Secondly, our findings could be influenced by internal and external conditions that might impact on the success of chronic care programs [46] and which were not considered in these models. For example, further studies could focus on the mediating or moderating effects of other governance tools on chronic care programs when they are applied in combination.

Findings regarding the CCM effect on overall satisfaction may be considered consistent with the inconclusive results found by Nolte et al. [6]. However, the results related to the null role played by CCM and education on the overall satisfaction and better self-management were surprising. For CCM, reviews based on administrative data found significant positive differences related to CCM programs only for some diseases [7], while no important differences emerged in terms of patients’ perception when considering (single and/or multiple chronic) patients as a whole..

This finding can be explained by the fact that after the first year of implementation (2010), GPs tended to align themselves with the average performance of their colleagues [35]. On the other hand, it could be explained by the fact that the regional government in Tuscany introduced indicators of the monitoring system for the chronic care programs, as well as health promotion indicators, into its PES for multi-layer providers: LHAs; districts and GPs. This means that even the GPs who did not adhere to CCM programs were evaluated on some indicators of the CCM monitoring system. Nuti et al. and Vainieri et al. reported that the Tuscan PES led to improvements in relation to a high number of indicators thanks to the integrated governance tools [39, 47]. On the basis of this evidence, we argue that the improvements introduced in Tuscany by these integrated governance tools clouded out the benefits coming from CCM program encouraging all GPs (not only those who adhered to the CCM program) to follow the core chronic care strategies.

## Conclusions

Most of the strategies that significantly affected overall satisfaction and the perception of better self-management seemed to be the same for both single and multiple chronic patients. While education did not influence the two outcomes, the monitoring strategy positively affects both of them. In fact, educational programs that generally have a positive effect, especially for single disease chronic patients, in our study were shown not have any. We argue that this depends on how the program is deployed by GPs: self-management programs follow precise rules and principles [34], while other studies have highlighted that the GP’s role may affect the success of these initiatives [21]. Our findings on monitoring strategies confirm the importance of such fundamental strategy in chronic management.

Relational continuity for both groups had a highly positive effect on better self-management. This confirms the pivotal role that the accumulated knowledge of GP plays in primary care in terms of patients’ perception [29]. The higher the relational continuity, the higher the probability of better self-management. However, relational continuity was mildly associated with overall satisfaction only for single chronic patients. This evidence suggests that multiple chronic patients may have different expectations.

An interesting finding of this study is that the CCM program designed and adopted in Siena seems not make any difference to either the overall satisfaction or better self-management for single and multiple chronic patients. One possible explanation lies in the fact that Tuscan PES systematically monitors some elements of chronic care management at all provider levels, independently of CCM enrolment, which may mask the effect of the CCM. In addition, another study argued that there is an isomorphic effect within GPs, so that differences between patients enrolled and not enrolled in CCM may decline because GPs tend to align their behaviours [35] irrespectively of the strategies. Hence, further investigations are needed to better understand the effects and role of multilayer strategies, such as global monitoring systems applied at different levels.

## Abbreviations

CCM: Chronic Care Model
DMP: Disease Management Program
GP: General Practitioners
LHA: Local Health Authority
PES: Performance Evaluation System
